# Supplementary material for: Single Cell Transcriptomic and Chromatin Profiles Suggest Layer Vb Is the Only Layer With Shared Excitatory Cell Types in the Medial and Lateral Entorhinal Cortex
Source: Front Neural Circuits. 2022 Jan 26;15:806154. doi: 10.3389/fncir.2021.806154 (PMC8826650; doi:10.3389/fncir.2021.806154)
Supplement: Supplementary file 14 [file Data_Sheet_1.ZIP › Inh_vs_Exc_Motifs/homerResults/motif3.similar.html]

motif3

## Information for motif3

C
G
T
A
A
T
C
G
A
C
G
T
A
C
T
G
G
T
C
A
A
G
T
C
G
T
A
C
A
G
C
T
G
A
C
T
T
C
A
G
  
Reverse Opposite:  

A
G
T
C
C
T
G
A
C
T
G
A
A
C
T
G
A
C
T
G
C
A
G
T
A
G
T
C
C
G
T
A
T
A
G
C
G
C
A
T
  

|  |  |
| --- | --- |
| p-value: | 1e-85 |
| log p-value: | -1.980e+02 |
| Information Content per bp: | 1.674 |
| Number of Target Sequences with motif | 2187.0 |
| Percentage of Target Sequences with motif | 17.86% |
| Number of Background Sequences with motif | 4532.6 |
| Percentage of Background Sequences with motif | 11.75% |
| Average Position of motif in Targets | 337.5 +/- 269.2bp |
| Average Position of motif in Background | 389.8 +/- 353.6bp |
| Strand Bias (log2 ratio + to - strand density) | -0.0 |
| Multiplicity (# of sites on avg that occur together) | 1.12 |
| Motif File: | file (matrix) reverse opposite |

### Similar de novo motifs found

|  |  |  |  |  |  |  |  |
| --- | --- | --- | --- | --- | --- | --- | --- |
| Rank | Match Score | Redundant Motif | P-value | log P-value | % of Targets | % of Background | Motif file |
| 1 | 0.942 | A G T C C A T G T C G A A C T G A C T G A C G T A G T C C T G A | 1e-81 | -187.207665 | 37.09% | 29.05% | motif file (matrix) |
| 2 | 0.778 | A G T C G T A C G T C A T C G A C A G T T C A G G T C A A G T C G A T C G A C T C G A T A C T G | 1e-36 | -83.829173 | 10.35% | 7.21% | motif file (matrix) |
| 3 | 0.873 | C A T G A G C T T A C G C T G A T G A C G A T C A G C T A C G T C T A G C T A G T C G A A G T C | 1e-33 | -77.706277 | 1.59% | 0.58% | motif file (matrix) |
| 4 | 0.769 | T G C A T G A C C T G A A C T G C T A G T C A G A G T C G T C A T G C A C G A T A C G T G C T A | 1e-32 | -75.062900 | 19.75% | 15.69% | motif file (matrix) |
| 5 | 0.750 | C A G T T A C G T G C A A G T C T G A C A G C T T G A C G C T A C A G T G C A T | 1e-30 | -70.916425 | 25.04% | 20.68% | motif file (matrix) |
| 6 | 0.825 | G T A C A C T G G A C T C A T G A G T C A G T C A G T C A G C T | 1e-26 | -61.694157 | 27.65% | 23.43% | motif file (matrix) |
| 7 | 0.720 | C T G A T C G A A C G T A C T G C G T A A G T C A G T C A T G C | 1e-25 | -59.437283 | 21.65% | 17.89% | motif file (matrix) |
| 8 | 0.715 | C G A T A C T G G T A C G A T C G A T C G A C T C G A T A T C G T G C A A G C T C G A T C A T G | 1e-22 | -50.687563 | 8.83% | 6.54% | motif file (matrix) |
| 9 | 0.671 | G C T A T A C G C A G T T A C G G T C A A T G C G A T C A T G C C A T G C T A G | 1e-21 | -49.043848 | 32.05% | 28.10% | motif file (matrix) |
| 10 | 0.654 | G C A T C A T G C G T A G T A C C G T A A G T C G C A T A T C G | 1e-16 | -38.099740 | 16.62% | 13.93% | motif file (matrix) |
| 11 | 0.683 | C A G T A T C G C T G A G A T C G T A C G A C T T G A C G T C A A C G T A G C T A G C T A G T C | 1e-16 | -37.909125 | 17.04% | 14.33% | motif file (matrix) |
| 12 | 0.673 | G T A C C G A T A T C G G C T A C G A T C A G T A C T G G C T A G A T C G T A C G C A T T G A C | 1e-14 | -33.158518 | 3.15% | 2.07% | motif file (matrix) |
